# Type 1 Pulmonary Hypertension and Silicosis in a Bluestone Cutter: A Case Report on Raising Awareness

**DOI:** 10.7759/cureus.35425

**Published:** 2023-02-24

**Authors:** Kunjal Luhadia, Kanica Yashi, Jaswinder Virk, Taral Parikh, Lokesh Goyal, Ahmad S Alam, Prabal Chourasia, Abigail Quintos

**Affiliations:** 1 Internal Medicine, Bassett Medical Center, Cooperstown, USA; 2 Cardiology, Bassett Medical Center, Cooperstown, USA; 3 Pediatrics, Hamilton Health Center, Harrisburg, USA; 4 Hospital Medicine, CHRISTUS Spohn Hospital Corpus Christi - Shoreline, Corpus Christi, USA; 5 General Surgery, John Hunter Hospital, Newcastle, AUS; 6 Hospital Medicine, Mary Washington Hospital, Fredericksburg, USA; 7 Pulmonary and Critical Care Medicine, Bassett Medical Center, Cooperstown, USA

**Keywords:** case report, awareness, hazard, hypertension, pneumoconiosis, silicosis, bluestone cutting, bluestone, pulmonary arterial hypertension, type 1 pulmonary hypertension

## Abstract

This case report describes a patient who developed pneumoconiosis in the form of silicosis and group 1 pulmonary hypertension (PH) due to his unprotected work as a bluestone cutter. Bluestone is a type of sandstone used in outdoor construction commonly in the North-east region of the US. In the literature and to our knowledge, blue stone mining has not been viewed as a risk factor for pneumoconiosis. This case report aims to increase awareness about this occupational hazard. Additionally, it is known that chronic silicosis with massive pulmonary fibrosis can lead to hypoxemia and group 3 pulmonary hypertension. This case, however, demonstrates a possibility of silica dust exposure leading to group 1 pulmonary arterial hypertension.

## Introduction

Bluestone is a type of sandstone found in South Central New York and North-Eastern Pennsylvania regions of the US and is commonly used in outdoor construction to pave patios, walkways, stairs, etc. Bluestone primarily consists of feldspars and quartz, which are a type of silicate [1]. Silicosis is a disease mostly associated with crystalline silica exposure. Crystalline silica is a common component of soil and rocks; consequently, workers in many industries and occupations are potentially exposed to it [2]. However, bluestone mining, which also exposes one to silica dust, to our knowledge has not been viewed as an occupational hazard potentially leading to pneumoconiosis.

The pathogenesis of silicosis involves oxidative damage caused by silica which results in the release of inflammatory cytokines, an increase in cell signaling, and apoptosis of parenchymal cells and macrophages. There is fibroblast hyperactivity, which causes an initial nodular pattern of interstitial lung disease that could then progress into massive pulmonary fibrosis [3,4].

Pulmonary artery hypertension (PAH) is a subtype of pulmonary hypertension caused by pulmonary arterial remodeling. Our patient was also found to have high pulmonary artery pressures and a picture of group 1 PAH out of proportion to his lung disease without hypoxemia. This brings one to hypothesize that silica dust exposure could lead to direct arterial damage and raise pulmonary artery pressures. There is data to suggest that ultra-fine silica particles may diffuse from the lungs across the pulmonary epithelium into the systemic circulation. Furthermore, the inflammatory mediators can affect the vascular endothelium leading to vascular injury. The recurring injury to the pulmonary vasculature may lead to the development of pulmonary hypertension [5].

## Case presentation

The patient is a 57-year-old male with a past medical history significant for interstitial lung disease and obstructive lung disease, who worked as a bluestone cutter for 30 years, and presented to the pulmonologist with symptoms of nonproductive cough for nine months and progressively worsening dyspnea. He was using umeclidinium bromide/vilanterol for three to four months without much relief. At the time of presentation, his vital signs included: a normal body temperature, blood pressure of 132/88, pulse rate of 64, respiratory rate of 12, and oxygen saturation (SpO2) of 98% on room air. On his physical examination, there was no cervical or supraclavicular adenopathy, and no jugular venous distension, lung examination revealed adequate air entry bilaterally, clear and equal breath sounds bilaterally, and no wheezing, crackles, or rhonchi. He did not have clubbing, cyanosis, or pedal edema. He, however, did have rough fingers and hands. He had worked as a bluestone cutter without any kind of protection for 30 years. His pulmonary function tests (PFTs) showed mild airflow obstruction (forced expiratory volume (FEV)1/forced vital capacity (FVC) 59, FEV1 30 L (86% predicted), FVC 4.37 L (77% predicted), no significant bronchodilator response, total lung capacity 86% predicted, diffusing capacity of lung for carbon monoxide (DLCO) 76% predicted), no overt restriction and normal diffusing capacity. His CT scan of the chest had shown findings of mediastinal and hilar adenopathy as well as middle to bilateral upper lobe parenchymal infiltration. It was suspected that he had either sarcoidosis or pneumoconiosis due to his line of work. 

He subsequently underwent bronchoscopy with endobronchial ultrasound-guided needle aspiration of mediastinal lymph nodes for histopathologic diagnosis. Lymph node biopsies revealed non-caseating granulomas which were negative for acid-fast bacilli (AFB) and fungal staining and cultures and absence of polarized foreign bodies (silica). These findings were suggestive of a diagnosis of sarcoidosis. He was started on 30mg of daily prednisone. After taking it for three weeks he had an improvement in symptoms including both dyspnea and cough. 

**Figure 1:**
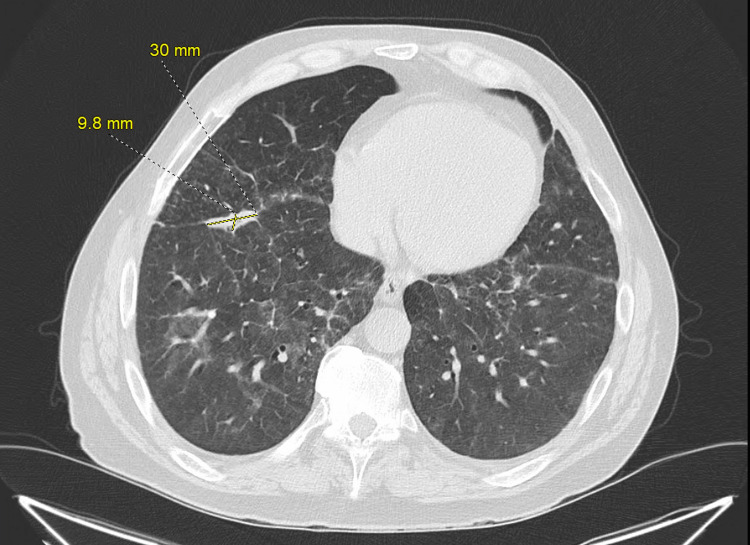
CT scan at eight months Lung nodule measuring 30 mm x 9.8 mm

**Figure 2:**
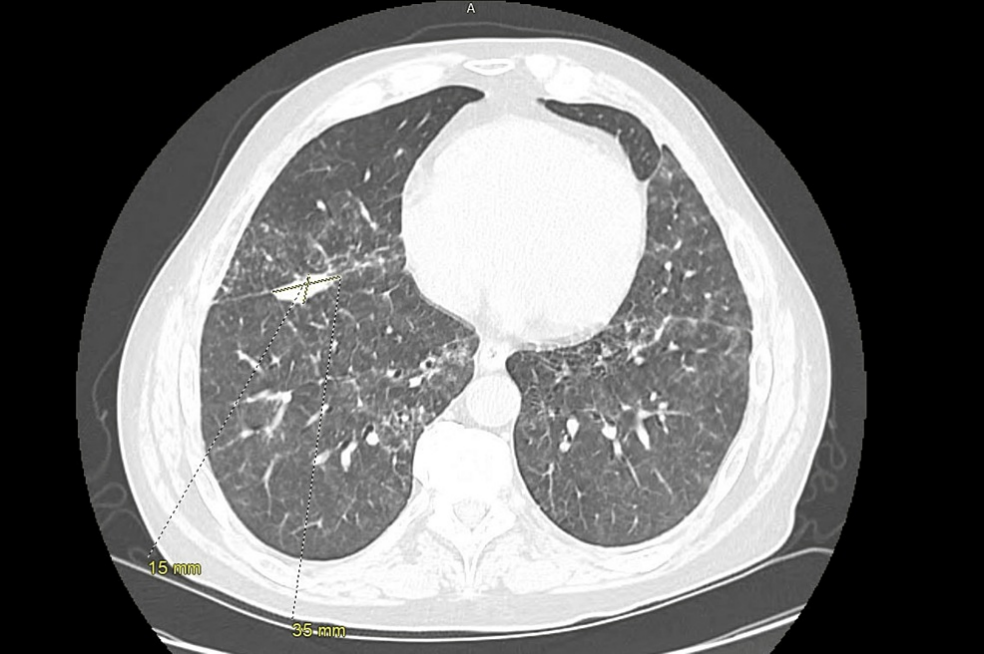
Worsening of the interstitial lung disease confirmed by diffuse increased interstitial nodularity A conglomerate lesion is present along the fissure on the right which appears to have progressed (measuring 35 mm x 15 mm) when compared to the previous examination which measured 30 mm x 9.8 mm.

However, eight months later the patient still had a cough despite a trial of steroids and inhaled bronchodilators (Figure 1) [6]. It was thought that the patient had steroid-resistant sarcoidosis. He was seen by a sarcoid specialist who recommended a repeat trial of oral steroids and if the symptoms were to improve, then methotrexate was recommended, if not, a determination could be made that anti-sarcoid medication would not work. However, the patient was unable to tolerate the steroids and they had to be discontinued. He was then started on methotrexate without much improvement and so this medication was stopped as well. At this time the CT scan of his chest showed a worsening of his interstitial lung disease (Figure 2). 

Around this time it was observed that other patients who also had a history of bluestone mining from the same region were presented with similar complaints [7]. Since our patient was not improving much with the use of steroids, we speculated that the patient had silicosis from the bluestone mining instead of sarcoidosis. The patient was advised to avoid any further exposure to silica and to stop mining bluestone.

**Figure 3:**
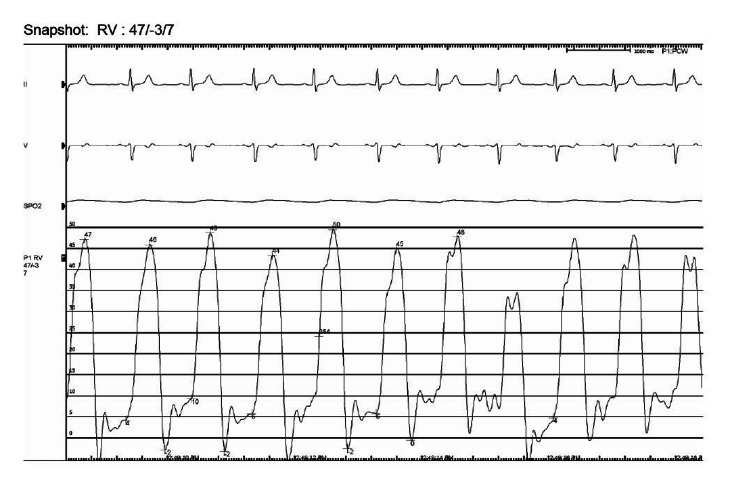
Elevated right ventricular pressure on right heart catheterization

Before he could wait and observe for any change in symptoms being away from work, the patient had a hospitalization for acutely worsening dyspnea and was found to be tremendously tachycardic with exercise. The patient was not hypoxic, not even on ambulation, with SpO2 remaining mid-90s, but heart rate raising to 150 to 160s just walking. An echocardiogram showed severe pulmonary hypertension, with a right ventricular (RV) systolic pressure >60mmHg and an approximate mean right atrial pressure in excess of 15 mmHg. The high pulmonary pressures seemed out of proportion to his lung disease without hypoxia, which is the underlying pathophysiology for pulmonary vasoconstriction in group 3 pulmonary hypertension (PH), hence a component of group 1 PAH was postulated. Group 4 PH was ruled out with a normal lung ventilation-perfusion scan. A right heart catheterization revealed a mean pulmonary arterial (PA) pressure of 28 mmHg against a normal wedge pressure of 10 mmHg. Right atrial pressure was normal at 5 mmHg. Pulmonary vascular resistance (PVR) was elevated. Right ventricular pressure was elevated up to 50 mmHg (Figure 3). Group 2 PH was ruled out with normal wedge pressure. With PVR being elevated, we were left with group 1 and group 3 PH, and that certain medications may have a role in treatment. 

Per the AMBITION Trial, the patient was started on ambrisentan and tadalafil, with improvement in symptoms. After about two months and intolerance issues, ambrisentan was discontinued, and he was kept on tadalafil monotherapy and diuretics. This regimen helped tremendously with his palpitations and tachycardia, but there was no significant change in his dyspnea. The patient continued to be followed at the pulmonary clinic and was taken out of work indefinitely due to his pneumoconiosis from bluestone mining. He was followed up at the pulmonary clinic six months later and demonstrated an improvement in his dyspnea as well as his RV pressure on transthoracic echocardiogram. 

## Discussion

This case brings to attention the potential respiratory hazard associated with bluestone mining. Inhaling silica dust from bluestone mining has been associated with the development of pneumoconiosis / particularly silicosis. This is further supported in a recent case series from our hospital’s pulmonary division that demonstrated 12 patients with similar occupational exposure developing hilar/mediastinal adenopathy, calcifications, reticulonodular opacities, and interlobar septal thickening. In this series, half the patients who underwent lymph node biopsy showed non-caseating granulomas suggestive of sarcoid-like reaction, one pathology suggestive of silicate, one with pulmonary fibrosis with mortality, and four cases showed features suggestive of progressive massive fibrosis [7].

Typically, workers involved with bluestone mining are not required to wear protective respiratory equipment as currently it is not viewed as a public health hazard. Like our patient, there are potentially other workers who suffer from silicosis and its debilitating symptoms. Since there is no cure nor any specific treatments for the disease, the emphasis should be on prevention as silicosis can be completely prevented with proper equipment and monitoring of exposure levels.

Group 1 PAH occurs when the arteries in the lungs become narrowed, thickened, or stiff. There are several causes of PAH which include: idiopathic, heritable, congenital heart disease, liver disease, HIV, connective tissue disorders, and certain drugs and toxins [8]. 

There is one study that showed the effects of exposure to crystalline silica in mice. The results of the study showed that silica-exposed mice showed signs of vascular remodeling including pulmonary artery muscularization, vascular occlusion, and medial thickening. It was also found that the expression of pro-inflammatory and pro-remodeling genes was significantly up-regulated, and the genes involved in the regulation of endothelial function were significantly attenuated [9]. 

Apart from silicosis as an interstitial lung disease potentially causing group 3 PH, further studies should be done to evaluate whether exposure to silica dust may have direct effects on the pulmonary vasculature leading to PAH.

## Conclusions

The most important aspect of this case is to raise awareness about the potential risk of developing pneumoconiosis with bluestone mining, and this should be viewed as a public health hazard. This is because unprotected work as a bluestone cutter leads to silicosis, and there is a possibility that it also leads to type 1 PH. There should be increased awareness about these findings as this disease is only preventable and not curable once the disease process takes place. Many times bluestone cutters are independently employed and they are completely unaware of the detrimental effects. For this reason, there should be increased awareness, and furthermore, Occupational Safety and Health Administration (OSHA) guidelines should be strictly followed at all bluestone-cutting/mining workplaces. Silicosis should be high on the differentials if a patient presents with a history of bluestone mining along with respiratory and radiographic findings of interstitial lung disease or sarcoidosis. Apart from the pulmonary parenchymal effects of silica dust exposure, further studies are encouraged to determine any direct effects on the pulmonary vasculature as well. 
